# Securing the Future: Strategies for Global Polio Vaccine Security Amid Eradication Efforts

**DOI:** 10.3390/vaccines12121369

**Published:** 2024-12-04

**Authors:** Vachagan Harutyunyan, Ann Ottosen, Rachel M. Burke, Derek Ehrhardt, Meredith Shirey, Rissa Durham, David Woods

**Affiliations:** 1 World Health Organization, 1211 Geneva, Switzerland; 2 UNICEF, 2150 Copenhagen, Denmark; 3 Gates Foundation, Seattle, WA 98109, USA; 4 Independent Researcher

**Keywords:** polio eradication, vaccine security, global OPV stockpile, immunization policy, inactivated poliovirus vaccine (IPV), oral poliovirus vaccine (OPV), vaccine production and distribution, global health security, poliovirus containment

## Abstract

**Background/Objectives:** As we commemorate 50 years of the Expanded Programme on Immunization (EPI), the global mission to eradicate polio stands at a critical juncture. While remarkable progress has been made over the past decades, ensuring a steady supply of polio vaccines remains a significant challenge that could undermine these achievements. This manuscript aims to address the complexities of polio vaccine security within the context of the Immunization Agenda 2030 (IA2030) and the Global Polio Eradication Strategy 2022–2029, proposing actionable strategies to strengthen the vaccine supply. **Methods:** This manuscript analyzes obstacles to vaccine security, including supply disruptions and market uncertainties. It presents the Polio Vaccine Security Framework as a key strategy for addressing these challenges. Data were gathered from Global Polio Eradication Initiative (GPEI) reports, consultations with key stakeholders, and analyses of past vaccine shortages. **Results:** The findings indicate that the primary risks to vaccine security include the lack of a coherent long-term policy framework on polio vaccination, the absence of a clear polio vaccine development roadmap, and insufficient long-term, predictable forecasting. Additionally, stronger coordination is needed between stakeholders involved in vaccine supply, polio containment, and research, as well as addressing challenges related to financing and access to resources. **Conclusions:** A robust, adaptable, and sustainable approach to vaccine security, proposed in the Polio Vaccine Security Framework, is critical to achieving and sustaining polio eradication. Collaboration among policymakers, manufacturers, and stakeholders to implement it is essential to ensure the uninterrupted supply of polio vaccines, protecting the progress made over the past half century, and preventing a resurgence of poliovirus in the future.

## 1. Introduction

The year 2024 marks the 50th anniversary of the Expanded Programme on Immunization (EPI), a monumental public health initiative that has been instrumental in reducing childhood mortality and combatting infectious diseases globally. Since its inception in 1974, the EPI has expanded access to life-saving vaccines, preventing millions of deaths and substantially lowering the global burden of vaccine-preventable diseases [1].

Building on the successes of the EPI, the Immunization Agenda 2030 (IA2030) sets forth a visionary framework for the next decade, focusing on universal access to vaccines and achieving equitable immunization coverage worldwide. Central to the IA2030 are the key pillars of supply and sustainability, and research and innovation. By highlighting the need for a reliable and uninterrupted supply of vaccines, the IA2030 aims to prevent shortages and uphold the gains achieved through disease control initiatives. This involves strengthening supply chains, enhancing production capabilities, and fostering international collaboration to maintain vaccine security [2]. Furthermore, the agenda emphasizes the importance of research and innovation to address emerging health challenges and improve vaccine efficacy and delivery methods [1].

Among the EPI’s many achievements, the global effort against polio stands out as one of the most significant public health victories. Thanks to the widespread use of polio vaccines, global wild polio cases have been reduced by more than 99% since 1988, from over 350,000 cases annually to just a handful of cases today, confined to oneregion. This success has brought the world to the brink of eradicating a disease that once caused widespread paralysis and death, particularly among children [3,4].

Polio eradication, however, cannot be viewed as a singular achievement, but rather as an ongoing commitment to sustaining progress. Central to this commitment is the concept of polio vaccine security, which aims to ensure the timely, sustained, and uninterrupted supply of appropriate types of affordable, quality-assured vaccines. As the world approaches polio eradication, ensuring an uninterrupted supply of polio vaccines remains critical—not only for achieving the final goal of global eradication but also for preventing a resurgence of the virus in a post-eradication world [5,6,7]. This is particularly important as countries face evolving epidemiological risks requiring the continued use of the OPV, while, at the same time, the global health community transitions away from OPV use toward the Inactivated Poliovirus Vaccine (IPV) to mitigate the risk of vaccine-associated paralytic poliomyelitis (VAPP) and vaccine-derived poliovirus outbreaks. The withdrawal of the trivalent oral polio vaccine (tOPV) in 2016 marked a significant step in this transition [8].

Maintaining polio vaccine security is thus pivotal to sustaining the successes achieved to date and protecting future generations from poliovirus. The cessation of certain polio vaccines, such as the trivalent OPV (tOPV) in 2016 and the Sabin monovalent OPV type 2 (mOPV2) between 2020 and 2023, along with upcoming transitions like the expected bOPV cessation, presents challenges. Addressing these challenges requires a strong, coordinated effort from national and international stakeholders to ensure an adequate global supply of vaccines for both routine immunizations and outbreak responses [6,7,9,10]. Past experiences with vaccine shortages, such as the IPV shortage in the aftermath of the global switch from the tOPV to the bivalent OPV (bOPV) in 2016, underscore the critical importance of having a reliable supply chain, adaptable production systems, and effective international cooperation [4,7,11].

In this context, polio vaccine security becomes not only a matter of achieving and maintaining the gains of polio eradication but also an important element of the broader framework of global health security. By ensuring that suitable polio vaccines of assured quality are always available, the global community can prevent any potential resurgence and safeguard the hard-won progress achieved through decades of effort. As we reflect on the 50-year journey of the EPI, the ongoing challenges and achievements in polio eradication remind us that vaccines are a powerful and critical tool in the fight against infectious diseases.

## 2. Background

**Figure 1:**
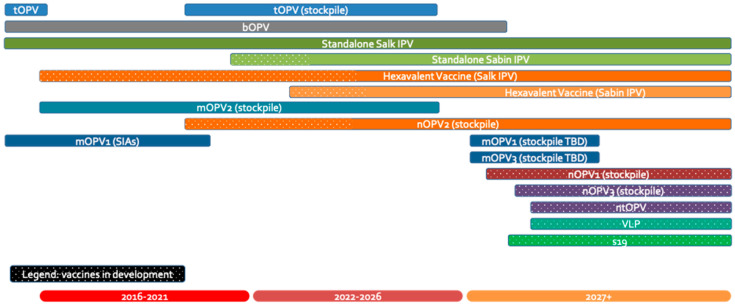
Current and future polio vaccines.

The landscape of polio vaccines is highly complex, as evidenced in Figure 1, which illustrates the various vaccine types in use and under development, their timelines, and the potential transitions between them. Over the past decades, it has undergone multiple transitions, including the shift from the trivalent oral polio vaccine (tOPV) to the bivalent OPV (bOPV), the introduction of the Sabin monovalent OPV2 (mOPV2) followed by the novel OPV2 (nOPV2), and the rollout of hexavalent IPV-containing vaccines. Each transition, driven by evolving epidemiological needs and public health strategies, has created opportunities while also posing challenges to maintaining a consistent vaccine supply.

A notable example is the IPV shortage following the global switch from the tOPV to the bOPV in 2016 [4,7,11]. The introduction of at least one dose of IPV was recommended to protect children from type 2 poliovirus-induced paralysis. However, global IPV production could not ramp up quickly enough to meet the sudden surge in demand, resulting in 28% of countries experiencing stockouts or delays in introducing the IPV [4]. Similarly, the Global OPV Stockpile, designed as an emergency reserve to combat Circulating Vaccine-Derived Poliovirus Type 2 (cVDPV2) after the switch, faced significant challenges in meeting demand. Initially, the rapid expansion of cVDPV2 outbreaks outpaced the capabilities of traditional consumption-based forecasting methods, leading to shortages. Later, following the introduction of the nOPV2 in 2020, reliance on a single supplier, coupled with production issues, resulted in prolonged periods of vaccine scarcity. These disruptions delayed responses, allowing the virus to spread unchecked in some cases [4,12,13,14]. These examples highlight the critical need for a more resilient vaccine supply system, capable of adapting to unforeseen production challenges to sustain eradication efforts.

Pricing is a critical factor, especially in low-resource settings, where affordability can impact access to essential immunization. Vaccine costs are shaped by substantial investments in research and development, production scale-up, and compliance with stringent manufacturing standards [1,6,11].

A major component of these production processes is the adherence to biosafety and biosecurity measures. Strict containment practices are required during vaccine manufacturing to prevent the accidental or intentional release of live poliovirus strains, which could threaten global eradication efforts. Investments in biosecurity infrastructure, high-containment laboratories, and training are crucial to meet these standards [9,15], thereby contributing to the overall cost of vaccine production.

Further complicating decision making is the fact that the slim profit margins on the OPV offer manufacturers less incentive to produce it compared to more profitable vaccines. The heavy reliance on a small number of manufacturers for the production of critical components, such as Sabin bulk types 1 and 3 and novel oral polio vaccines (nOPVs), heightens the risk of supply chain disruptions. This dependence results in a delicate supply network, susceptible to significant impacts from unforeseen production or regulatory challenges, as demonstrated by the shortages experienced with the Inactivated Poliovirus Vaccine (IPV) and nOPV2 [4,10,15,16].

Longstanding uncertainties surrounding polio eradication timelines have led to ever-evolving polio vaccination policies, resulting in a complex and unpredictable market for polio vaccines. To address this problem, it is essential to develop a well-defined, consolidated long-term vaccination policy framework and a polio vaccines roadmap. This framework should clearly specify the necessary vaccines, their characteristics, timelines for introduction and withdrawal, and conditions for use. Providing such clarity would enable manufacturers to make informed decisions, facilitating effective long-term planning within the vaccine industry.

Addressing these multifaceted challenges requires close coordination among the following three critical operational areas: vaccine supply, research and product development, and poliovirus containment. Synchronizing efforts across policy and normative work, communication, and access to resources within these domains is essential to ensure that vaccine production aligns with public health needs, that innovative vaccines are developed and brought to market efficiently, and that containment measures are effectively implemented to prevent virus resurgence. This collaboration is vital not only for sustaining a healthy vaccine market but also for achieving the ultimate goal of global polio eradication [1,2,6,9,14,15].

### Origins and Development of the Polio Vaccine Security Framework

**Figure 2:**
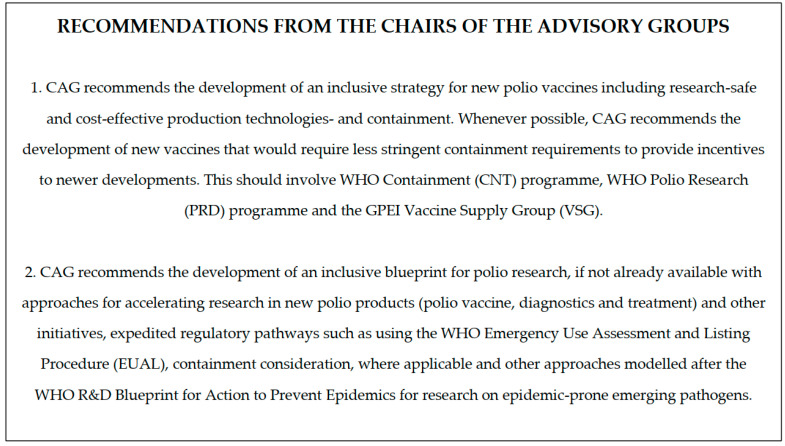
Recommendations for the chairs of the advisory groups, as outlined in the Report from the Sixth Meeting of the Poliovirus Containment Advisory Group (CAG6).

The development of the Polio Vaccine Security Framework was initiated in response to the growing need for a long-term, strategic approach to securing a reliable and uninterrupted supply of polio vaccines. This process began following recommendations from key advisory groups to the Global Polio Eradication Initiative (GPEI), including those from the Global Certification Commission (GCC) and the Containment Advisory Group (CAG), which were issued during the Sixth Meeting of the Poliovirus Containment Advisory Group (CAG6). Additionally, the Independent Monitoring Board (IMB) reflected its recommendations in its 22nd report [17]. These groups emphasized the critical importance of a coherent vaccine strategy (Figure 2) [17,18] aligning the vaccine supply, containment, and research efforts to ensure polio vaccine security during the eradication process and beyond.

The framework’s development was overseen by a dedicated steering team consisting of representatives from the GPEI’s Vaccine Supply Group (VSG), the Polio Research and Analytics Group (PRAG), and the Containment Management Group (CMG). This multidisciplinary team worked collaboratively to integrate insights from various operational areas, ensuring that the framework addresses the challenges posed by shifting vaccine needs and the requirement to meet poliovirus containment standards.

Shaping the framework were insights gathered during the Twenty Second Annual Consultation between the GPEI, vaccine manufacturers, National Authorities for Containment (NACs), and National Regulatory Authorities (NRAs). This consultation provided an important platform for dialogue, where key stakeholders emphasized the need for robust demand forecasting, a clear regulatory and normative framework, sustainable financial mechanisms, and support in implementing containment strategies.

Following this, additional consultations were carried out within the GPEI, as well as WHO and UNICEF regional offices, to incorporate both global and regional perspectives. Through this consultative approach, the framework was shaped to address the evolving challenges in vaccine supply, research, and containment, ensuring long-term security and readiness.

## 3. Introducing Polio Vaccine Security Framework

As highlighted in the previous sections, achieving global polio eradication demands a secure, sustained, and uninterrupted supply of vaccines. The Polio Vaccine Security Framework was developed by the GPEI to address the complex challenges associated with the vaccine supply and to ensure long-term security. This framework seeks to secure the availability of affordable, quality-assured vaccines both for poliovirus eradication and to protect the gains of the global eradication efforts in the post-certification period.

**Figure 3:**
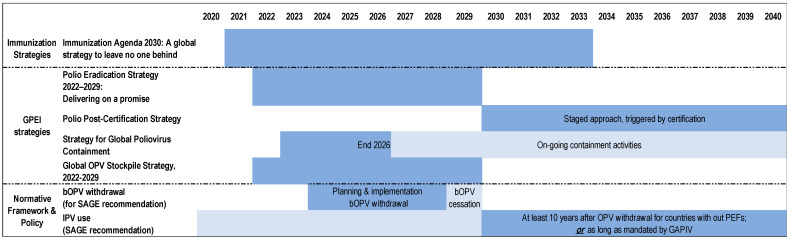
Mapping of strategies and policies related to Polio Vaccine Security (note: timelines presented as applicable at the time of drafting this framework; some timelines are under revision and will be noted in future versions).

The Polio Vaccine Security Framework is built on the following five key principles that form the foundation for achieving its objectives:

1. Interdependence of Operational Areas: The framework emphasizes the interconnected nature of the vaccine supply, research and development, and poliovirus containment. These elements influence one another, and their coordination is crucial for overall success. The alignment of activities in these areas ensures the availability of suitable vaccines and the safe and secure handling of polioviruses.
2. Long-Term Strategic Planning: The framework shifts the focus from reactive, short-term approaches to long-term strategic planning. A comprehensive vaccine policy and roadmap, supported by clear demand forecasts and product transitions, to be developed within the scope of the framework, will provide essential visibility for stakeholders, ensuring that production and supply can be aligned with global eradication goals.
3. Building on Existing Frameworks: The Polio Vaccine Security Framework complements and enhances existing policies and strategies (Figure 3), including the Immunization Agenda 2030 (IA2030), the Polio Eradication Strategy 2022–2026, and the Global Poliovirus Containment Strategy (Figure 2). By aligning with these frameworks, the initiative aims to foster a unified, effective approach to maintaining a polio-free world.
4. Sustainable Market for Polio Vaccines: Ensuring a sustainable market for polio vaccines is essential. This involves incentivizing manufacturers to continue production, particularly during transition periods or market uncertainty. Risk-sharing mechanisms, financial support for product development, and transparent communication of long-term demand are critical to maintaining engagement from manufacturers and ensuring a healthy, competitive market.
5. Ongoing Monitoring and Adaptation: Given the evolving nature of polio eradication and the vaccine landscape, the framework stresses the importance of continuous monitoring and adaptation. Regular updates and revisions will be necessary to respond to new challenges, ensuring the framework remains effective in securing the vaccine supply.

### 3.1. Goal and Objectives

The goal of the Polio Vaccine Security Framework is to ensure the timely, sustained, and uninterrupted supply of affordable, quality-assured polio vaccines. Achieving this goal is critical to the success of global polio eradication efforts and to preventing the re-emergence of poliovirus in the future.

#### 3.1.1. The Framework Outlines Three Primary Objectives

1. Strengthened Communication, Coordination, and Advocacy: Foster enhanced communication and coordination between global health stakeholders, manufacturers, and policymakers to ensure that vaccine security challenges are addressed comprehensively and strategically.
2. Development of a Coherent Normative and Policy Framework: Establish a unified policy framework that supports the long-term use of polio vaccines. This includes aligning vaccine supply and development efforts with containment and research requirements, ensuring that global standards and guidelines are adhered to across all areas from research to production and distribution.
3. Improved Financing and Access to Resources: Identify and mitigate financial risks by securing sustainable resources for vaccine production and distribution. This includes ensuring access to financing across key areas such as supply, containment, research, and development to support uninterrupted vaccine availability.

By integrating these objectives, the Polio Vaccine Security Framework creates a comprehensive strategy that strengthens the global capacity to prevent vaccine shortages, supports manufacturers’ efforts, and ensures that the infrastructure required for polio eradication remains robust and adaptable.

#### 3.1.2. Elements of the Polio Vaccine Security Framework

**Figure 4:**
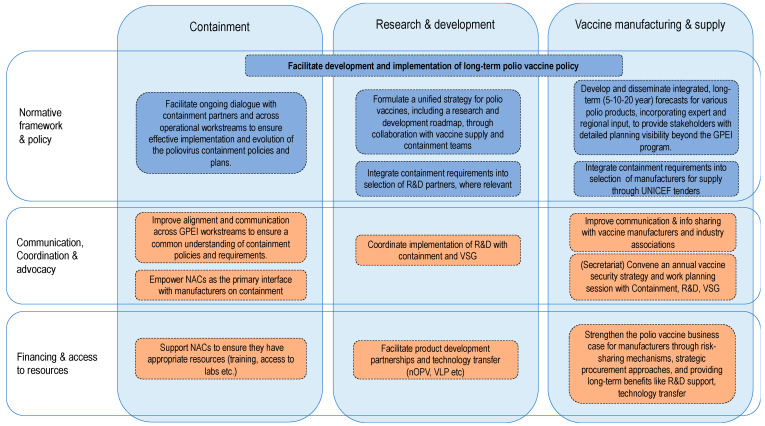
Key elements of the Polio Vaccine Security Framework.

The Polio Vaccine Security Framework is organized around six key elements, designed to ensure a holistic, sustainable approach to maintaining the supply of polio vaccines. These elements include three operational workstreams—vaccine supply, research and product development, and poliovirus containment—along with the following three cross-cutting themes: normative work and policy development, communication and advocacy, and financing and access to resources (Figure 4). Together, they form an interconnected structure that supports the long-term success of global polio eradication efforts.

##### Operational Workstreams

1. Vaccine Supply: Ensuring a continuous supply of vaccines is fundamental to the polio eradication effort. This involves robust forecasting, efficient production, procurement strategies, and stockpiling. It also means strengthening the supply chain to avoid disruptions. The Vaccine Supply Group (VSG) is tasked with overseeing this process, working closely with manufacturers and stakeholders to secure both OPVs and IPVs, which are critical for sustaining immunization efforts during and after eradication.
2. Research and Product Development: As we progress towards and beyond polio eradication, new products and innovations will be required to meet the evolving public health needs. This operational area focuses on the development of new and improved polio vaccines and other products to address emerging challenges and ensure long-term vaccine security; these include next-generation OPVs (nOPV1, nOPV3, and trivalent nOPV), the Sabin-based IPV (sIPV), the attenuated Sabin strain 19 (S19) vaccine, virus-like particles (VLPs), lower-cost hexavalent vaccines (Figure 1), and therapeutic options for the iVDPV. The GPEI partners coordinate closely in this domain to ensure alignment with long-term vaccine security goals, while also adapting to scientific and technological advancements.
3. Poliovirus Containment: Poliovirus containment is critical to minimize the risk of a facility-associated release of poliovirus, such as manufacturers and laboratories. The unintentional or intentional release of poliovirus could result in the re-establishment of poliovirus transmission, particularly in the post-OPV phase. This involves stringent safe and secure measures in laboratories, vaccine production sites, and other facilities that retain polioviruses. It involves implementing biorisk management policies and standards, as outlined the WHO Global Action Plan for Containment, 4th edition 2022 (GAPIV) [9]. It also includes a globally harmonized and rigorous conformity assessment process for facilities retaining poliovirus.

##### Cross-Cutting Themes

1. Normative Work and Policy Development: A clear and adaptable policy environment is crucial for guiding the strategic direction of all three operational areas. This includes establishing a long-term polio vaccination policy framework that outlines the types of vaccines needed, timelines for introduction and withdrawal, and how these decisions align with evolving research and development, containment requirements, and global eradication goals. There is a need for greater clarity and collaboration on several key aspects, including the bOPV cessation strategy, regulatory pathways for novel vaccines, long-term IPV strategies, and the application of containment standards in vaccine development and production. This clarity is essential for enabling manufacturers to make informed decisions about production capacity, investments in new technologies, and engagement in the polio vaccine market, ultimately contributing to a more secure and sustainable vaccine supply.
2. Communication and Advocacy: Coordinated communication among global stakeholders, governments, and vaccine manufacturers is vital for maintaining vaccine security. This includes fostering dialog between GPEI workstreams, vaccine manufacturers, national regulatory authorities, and other relevant organizations to ensure aligned priorities, consistent messaging, and efficient resource allocation. The existing communication platforms, such as the annual GPEI consultation with manufacturers and the UNICEF Vaccine Industry Consultation, provide valuable platforms for this collaboration. However, there is a need to enhance these platforms and expand communication efforts to ensure greater transparency, share long-term demand forecasts, and provide manufacturers with clear insights into the evolving policy landscape, fostering trust and enabling them to make informed decisions about their engagement in the polio vaccine market.
3. Financing and Access to Resources: This area of work emphasizes the need for predictable financing to mitigate risks to the polio vaccine industry and strengthen the business case for vaccine production. Key to this approach are risk-sharing mechanisms that help manufacturers maintain production during periods of uncertainty, such as the withdrawal of the bivalent oral polio vaccine (bOPV). These mechanisms reduce the financial burden on manufacturers, ensuring that production continues even when future demand is unclear.

Additional mechanisms such as financial support for product development and technology transfer will remain important to keep the polio vaccine pipeline dynamic and absorb the vaccine R&D costs. Long-term demand forecasting, offering visibility over 5 to 20 years, is another essential tool for ensuring that manufacturers can plan for sustained production. By creating a more predictable market environment and providing financial stability, the framework encourages continued investment in polio vaccine production, safeguarding the supply in the post-eradication era.

These six elements are not only complementary but highly interdependent. The effectiveness of each operational workstream is bolstered by the cross-cutting themes, creating a comprehensive strategy that ensures long-term polio vaccine security.

## 4. Discussion

As the global health community stands on the cusp of eradicating polio, ensuring polio vaccine security is even more critical than before. The sustained availability of necessary types of affordable, quality-assured vaccines is not only essential to achieving global eradication but also to maintaining a polio-free world in the post-eradication era [19]. The Polio Vaccine Security Framework aims to address both the present and future needs of the vaccine supply chain. The framework provides an umbrella strategy to mitigate the challenges to the polio vaccine security through fostering closer engagement among the vaccine supply, research and product development, and poliovirus containment.

### 4.1. Present and Future Challenges

Several significant challenges persist in the ongoing fight against poliovirus. The fragmented approaches and short-term planning within the GPEI and among stakeholders jeopardize long-term vaccine security. For example, product-specific and short-term forecasting, often linked to procurement cycles, restricts manufacturers’ visibility and hampers their capacity planning efforts. Uncertainties within the normative and policy framework, especially concerning the timeline for bOPV cessation and the future role of nOPVs post-bOPV, complicate manufacturers’ long-term investment decisions. Financial risks for manufacturers abound due to uncertain future demand and potential supply chain disruptions.

As a result, the vaccine market remains susceptible to supply disruptions driven by evolving timelines and fluctuating demand for various vaccine types like OPVs and IPVs. Against this background, there is hesitance on the part of vaccine manufacturers to commit to long-term production investments without a clear business case. Relying on a limited number of manufacturers for critical vaccine components further weakens the supply chain, creating vulnerabilities should market fluctuations or production bottlenecks occur.

The requirement to achieve safe and secure poliovirus containment is a necessary part of polio eradication and the post-eradication period. However, there is a current need for a stronger collaboration on the implementation of containment measures while effectively conducting supply and research activities. The intricate task of balancing global supply with containment requirements, especially in laboratories and production environments, highlights the need for coordinated strategies [15].

### 4.2. Opportunities Through the Polio Vaccine Security Framework

The Polio Vaccine Security Framework offers a strategic opportunity to address these challenges and secure a sustainable, long-term supply of vaccines. The framework’s focus on a coordinated vaccine supply, research and development, and poliovirus containment ensures that these operational areas work in tandem, mitigating the risks of supply shortages while fostering innovation in vaccine development.

There is broad recognition that a reliable and appropriate vaccine supply is a critical factor for the success of the eradication program. Without a steady and dependable supply of vaccines, efforts to control and eventually eradicate poliovirus will be jeopardized, potentially threatening the substantial progress made thus far.

Another significant opportunity lies in the recognition that securing the polio vaccine supply is not a task that can be achieved by supply or procurement agencies alone. It requires multi-sectoral coordination guided by a coherent normative and policy framework. This collaborative approach ensures that all facets of vaccine security are addressed, from product development and scale-up, and production and distribution to regulatory compliance and public health policies.

Another opportunity lies in leveraging existing platforms and structures that are already in place to address polio vaccine supply issues. For instance, the GPEI has well-established structures and processes that can be utilized to enhance the coordination and implementation of the polio vaccine supply.

Additionally, the Annual Consultation platform, which brings together GPEI stakeholders, the vaccine industry, and national regulatory and containment authorities, presents a valuable opportunity for fostering collaboration and alignment. By building on these existing mechanisms, the Polio Vaccine Security Framework can enhance its effectiveness and efficiency, ultimately contributing to a more robust and resilient vaccine supply system.

### 4.3. Implementation and Adaptability

The success of the Polio Vaccine Security Framework hinges on several of the following factors:

First, it needs to be effectively implemented and managed, with clear lines of responsibility and accountability. The GPEI Vaccine Supply Group (VSG) will play a critical role in coordinating these efforts, ensuring alignment between the framework’s objectives and the work of other GPEI workstreams and partners.

Second, the successful implementation of the Polio Vaccine Security Framework relies on its ability to remain adaptable to the changing landscape of polio eradication. As new scientific advancements are made and epidemiological risks evolve, the framework must respond dynamically, offering clear direction to both the polio vaccine industry and the GPEI partnership. Continuous monitoring and regular updates to the framework will ensure that it remains responsive to emerging challenges, such as new vaccine technologies, containment protocols, and changing demand patterns.

Furthermore, the framework’s integration of regional and global perspectives, through ongoing consultations with WHO and UNICEF regional offices, ensures that it addresses the specific needs of different regions. The coordinated efforts of these stakeholders, supported by clear communication channels and transparent decision-making processes, will be critical in aligning global vaccine supply with eradication goals. 

## 5. Conclusions

As the global health community approaches polio eradication, ensuring a consistent and uninterrupted supply of polio vaccines is critical to achieving and maintaining a polio-free world. The Polio Vaccine Security Framework is a robust strategy that aligns with the broader goals of the Immunization Agenda 2030 (IA2030) and the Polio Eradication Strategy 2022–2026 (2029). These global strategies emphasize the need for strong, resilient vaccine supply systems that are responsive to evolving epidemiological challenges.

The Polio Vaccine Security Framework thus plays a key role in safeguarding the progress made through decades of immunization efforts under the EPI. By addressing the complexities of vaccine supply, containment, and research, the framework helps ensure that future generations are protected from poliovirus re-emergence. Its integration with the IA2030 goals of equity, sustainability, and innovation in immunization provides a broader context for ensuring that the lessons learned from polio eradication contribute to strengthening global vaccine security for all vaccine-preventable diseases.

As we move into the post-eradication phase, the continued implementation of this framework, in coordination with the IA2030 and the GPEI partnership, will be vital to preventing any resurgence of polio and to maintaining global health security. The long-term planning, financing, and adaptability embedded in the Polio Vaccine Security Framework will not only support polio eradication, but also provide a model for securing vaccines for other global immunization efforts, marking a significant step toward a healthier, more resilient world.

## Data Availability

The data presented in this study are available within the article.
